# PUP-Fuse: Prediction of Protein Pupylation Sites by Integrating Multiple Sequence Representations

**DOI:** 10.3390/ijms22042120

**Published:** 2021-02-20

**Authors:** Firda Nurul Auliah, Andi Nur Nilamyani, Watshara Shoombuatong, Md Ashad Alam, Md Mehedi Hasan, Hiroyuki Kurata

**Affiliations:** 1 Department of Bioscience and Bioinformatics, Kyushu Institute of Technology, 680-4 Kawazu, Iizuka, Fukuoka 820-8502, Japan; Firdana.525@gmail.com (F.N.A.); nurnilamyani.514@gmail.com (A.N.N.); hasan.md-mehedi922@mail.kyutech.jp (M.M.H.); 2 Center of Data Mining and Biomedical Informatics, Faculty of Medical Technology, Mahidol University, Bangkok 10700, Thailand; watshara.sho@mahidol.ac.th; 3 Tulane Center for Biomedical Informatics and Genomics, Division of Biomedical Informatics and Genomics, John W. Deming Department of Medicine, School of Medicine, Tulane University, New Orleans, LA 70112, USA; malam@tulane.edu; 4 Japan Society for the Promotion of Science, 5-3-1 Kojimachi, Chiyoda-ku, Tokyo 102-0083, Japan

**Keywords:** pupylation, feature encoding, chi-squared, machine learning

## Abstract

Pupylation is a type of reversible post-translational modification of proteins, which plays a key role in the cellular function of microbial organisms. Several proteomics methods have been developed for the prediction and analysis of pupylated proteins and pupylation sites. However, the traditional experimental methods are laborious and time-consuming. Hence, computational algorithms are highly needed that can predict potential pupylation sites using sequence features. In this research, a new prediction model, PUP-Fuse, has been developed for pupylation site prediction by integrating multiple sequence representations. Meanwhile, we explored the five types of feature encoding approaches and three machine learning (ML) algorithms. In the final model, we integrated the successive ML scores using a linear regression model. The PUP-Fuse achieved a Mathew correlation value of 0.768 by a 10-fold cross-validation test. It also outperformed existing predictors in an independent test. The web server of the PUP-Fuse with curated datasets is freely available.

## 1. Introduction

Pupylation is a type of prokaryotic ubiquitin-like protein (Pup), which contributes to many cellular processes [1,2]. The Pup process connects the lysine residue with isopeptide bonds, called pupylation, which plays an important role in controlling signal transduction and protein degradation in prokaryotic cells [3,4]. Pup proteins tag intrinsically disordered and misfolded proteins to be degraded [3,5]. While pupylation and ubiquitylation are analogs in terms of function, they have different enzymologies [6]. Unlike ubiquitylation, pupylation involves two types of enzymes: deamidase of Pup (DOP) and proteasome accessory factor A (PafA) [3,7,8,9,10]. On the other hand, enzymes of pupylation are initiated from microbial species and exhibit no homology to ubiquitylation enzymes [7,11].

To know the molecular mechanisms of pupylation, it is necessary to define the substrates of pupylation and its sites precisely. Typically, this task is labor-intensive and time-consuming because of the large-scale analysis of proteomics; thus, a few computational methods of predicting pupylation sites have been proposed [12,13,14,15,16]. Liu et al. first developed the GPS-PUP predictor for the prediction of pupylation sites by the group-based prediction system (GPS) method [17]. Tung et al. presented the iPUP predictor that implemented the support vector machine (SVM) algorithm with the composition of pairs of k-space amino acids (CKSAAP) [18]. Chen et al. developed the PupPred predictor based on SVM [19], where the pairing of amino acids was used to encode the lysine-centered peptides. Recently, Hasan et al. proposed a web server, called pbPUP, to predict the pupylation sites using the profile-based features [20]. The predictors of GPS-PUP, iPUP, and pbPUP showed reasonable performance of population site prediction. However, when they were given higher specificity, their sensitivity score was low.

**Figure 1:**
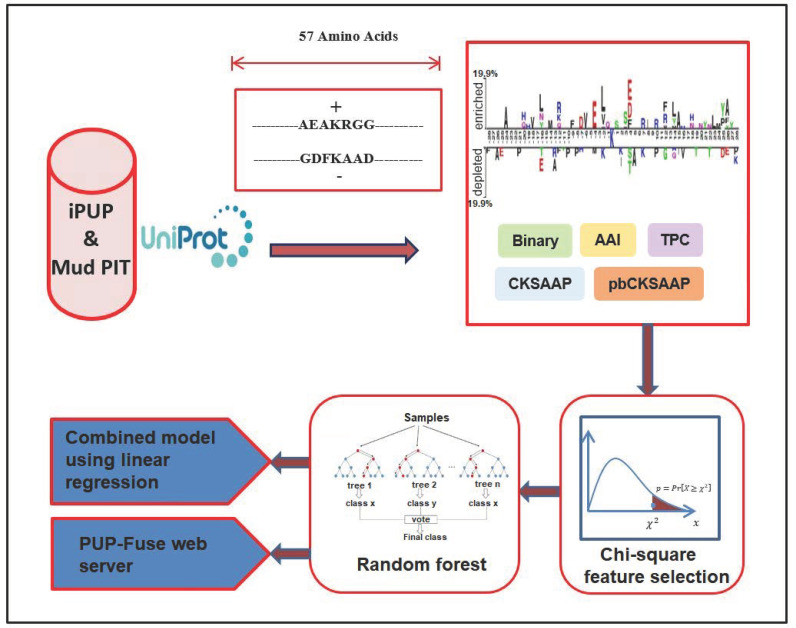
An overview of the proposed PUP-Fuse predictor.

In this study, we developed the PUP-Fuse as a machine learning (ML)-based predictor, as shown in Figure 1. In brief, we employed the PupDB database [21] to compile the positive and negative samples with a full sequence, encoded the sequence windows into numerical feature vectors by using multiple sequence encoding schemes, selected informative features, and inputted them to ML models. The PUP-Fuse integrated the multiple ML scores generated by the different, single encoding-employing ML methods to enhance the prediction performance.

## 2. Results and Discussion

### 2.1. Sequence Preference Analysis

**Figure 2:**
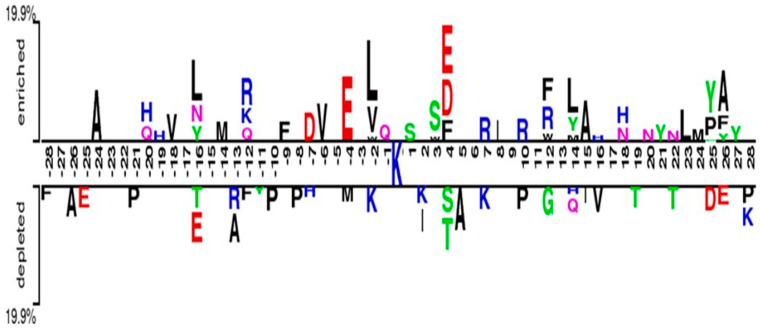
Two sample logo of the pupylation sites.

To extract the local sequence around prediction sites, we used a local sliding window consisting of 57 residues (−28~K~28). We used the two-sample logo [22] to display every 28 residues located upstream and downstream in the protein sequence with the pupylation site, as shown in Figure 2. Significant variances in the nearby pupylated sequences were found between the pupylation and non-non-pupylation sites. Particularly, residues “R, Y, and L” were more frequently observed in the enriched positions. In the depleted position residues, “P and K” were more frequently observed. On the other hand, no amino acid residues are stacked at some over- or under-represented positions of the surrounding sequences. For instance, at the enriched positions of −28, −27, −26, −25, −23, −22, −21, −17, −15, −13, −11, −10, −8, −5, −3, +2, +5, +6, +9, +11, +13, +17, +19, and +28 no stacked residues were found. Similarly, in the depleted position of −27, −24, −23, −22, −20, −19, −18, −17, −15, −14, −9, −6, −5, −3, −1, +1, +3, +6, +8, +9, +11, +13, +17, +18, +20, +21, +23, +24 and +27 no stacked residues were identified, suggesting significant information between the positive and negative samples. The above results indicate that a combination of the frequency- and position-based sequence encoding schemes is effective in identifying pupylation sites.

### 2.2. Performance Results on Training Dataset

**Figure 3:**
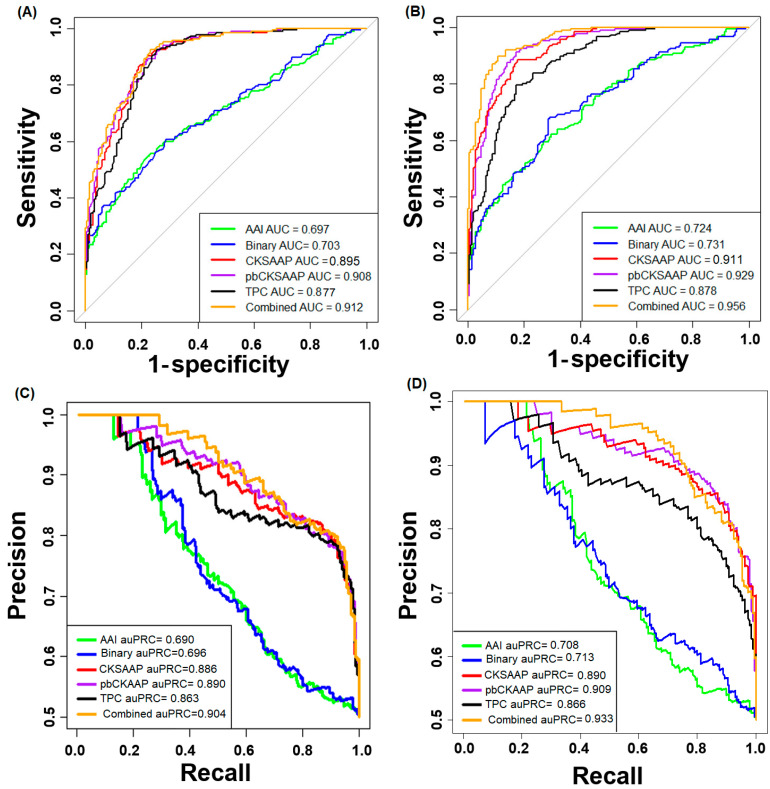
Prediction performance of the single encoding-employing models and their combined model (PUP-Fuse) on the training datasets. (**A**) No feature selection is employed. (**B**) Feature selection is used. (**C**) Precision vs. recall curve without feature selection. (**D**) Precision vs. recall curve with feature selection.

We have developed the five single encoding-employing random forest (RF) models and the combined model (PUP-Fuse) by linearly combining them. The PUP-Fuse optimized the five weight coefficients for the AAI, Binary, tripeptide composition (TPC), Profile-Based Composition of K-Spaced Amino Acid Pairs (pbCKSAAP), and CKSAAP-employing RF models as 0.01, 0.1, 0.3, and 0.3, 0.2, respectively. We evaluated the Sens, Spec, Acc, MCC, AUC values of the single encoding-employing RF models and the PUP-Fuse without any feature selection by 10-fold CV test, as shown in Table 1. The ROC and corresponding auPRC curves are shown in Figure 3A,C. The five measures of the PUP-Fuse were higher than those of any single encoding-employing RF model. The PUP-Fuse achieved a very high AUC of 0.913 and outperformed all the single encoding-employing models, which significantly outperformed all the single encoding-employing models with a two-sample *t*-test at *p*-value < 0.05 (Table 1).

The PUP-Fuse predictor was developed based on the PupDB database, where a positive-to-negative ratio of ~1:12 was highly imbalanced. Since the prediction accuracy of ML algorithms is seriously impaired by such unbalanced datasets [19,20], many site predictors of PTM use a fairly balanced ratio of positive to negative samples to train classification models [20,23,24]. On the training dataset, we compared the prediction performance (AUC) between 1:1, 1:2, and 1:all ratios of positive to negative samples (Figure S1). Since a 1:1 ratio provided a higher AUC value, we determined a 1:1 ratio as the optimal one. The size of the window is also an important factor to discriminate the positive sites from the negative ones. Based on AUC values, the window size was searched from 25 to 61 (Figure S2). An optimal window size of 57 was obtained because the AUC increasing rate from 45 to 57 was very low.

To investigate the validity of a high cutoff similarity of 80%, employed by CD-HIT [25], we compared the prediction performance with a cutoff of 80% to that with an ordinary cutoff of 30%. A cutoff of 30% produced the training dataset that contained 129 proteins with 141 pupylation and 141 non-pupylation sites (with a 1:1 positive-to-negative ratio). The overall performance of PUP-Fuse with a cutoff similarity of 30% a little decreased (AUC = 0.903) (Table S1) by the 10-fold cross-validation, but a cutoff similarity of 80% presented almost similar performance with a cutoff similarity of 30%.

### 2.3. Performance Optimization by Chi-Square Test

The proposed method describes some sniping sequence patterns for a pupylation site in a comprehensive way, while it results in a high-dimensional vector. Some redundant or irrelevant attributes may be present that affect accuracy reduction. Thus, we selected the informative features out of many features using a well-established chi-squared test. For each employed scheme, different feature subsets were selected, which contained the top-ranked features ranging from the top 20 to the top 500 with an interval of 20. All these curated feature subcategories were inputted to RF separately, and their respective performances were evaluated using 10-fold cross-validation (Figure S3). To end, the feature subset that reached the highest AUC was selected as the optimal one. In this approach, we selected the 260-, 100-, 200-, 240-, and 350-dimensional features from pbCKSAAP, AAI, Binary, CKSAAP, and TPC encodings, respectively. In the PUP-Fuse, the weight coefficients for the AAI, Binary, TPC, and pbCKSAAP-employing RF models were optimized as 0.1, 0.1, 0.3, 0.3, and 0.2, respectively. As shown in Figure 3B,D, the PUP-Fuse with the chi-squared test achieved higher AUC and auPRC values than that without the feature selection. The PUP-Fuse with feature selection achieved an accuracy of 88.4% (Sn = 88.1% and MCC = 0.768) at a specificity control of 88.1% on the training data (Table 2). The PUP-Fuse reached a remarkable AUC value of 0.956, which significantly outperformed all the single-employing-based models with a two-sample *t*-test at the level of *p*-value < 0.05 (Table 2).

### 2.4. Comparison among Different ML Methods on Training Dataset

**Figure 4:**
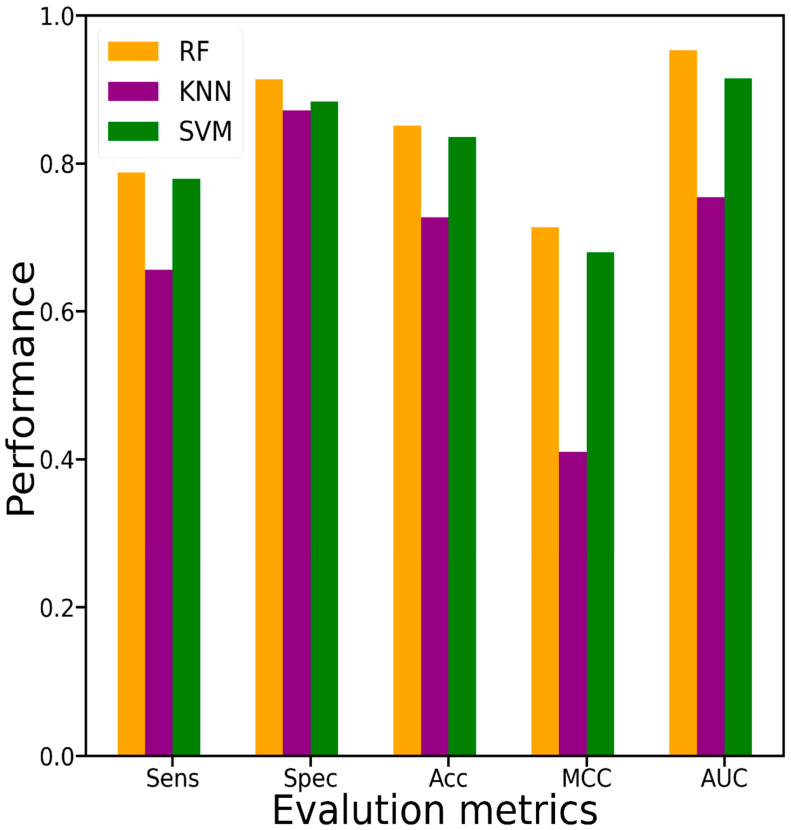
Performance comparison among three different machine learning (ML) methods of RF, SVM and KNN.

Selecting an optimal ML method is an essential step. Therefore, to verify the effectiveness and superiority of the RF algorithm employed by the PUP-Fuse, we compared it with the KNN and SVM algorithms on the same training dataset by a 10-fold CV test. In order to make a fair comparison, the KNN and SVM models implemented the same encoding schemes as the PUP-Fuse. As shown in Figure 4, the RF model yielded a higher AUC than the other two ML models, which was approximately 2–5% higher than the AUCs of the other models.

### 2.5. Comparison of PUP-Fuse with Existing Methods on Independent Dataset

Several computational methods had been proposed for the prediction of pupylation sites. In order to compare the PUP-Fuse with the four existing methods (GPS-PUP, iPUP, PUPS, and PbPUP), an independent dataset of 86 pupylation sites from 71 pupylated proteins and 1136 non-pupylation putative sites was used. Even though the PUP-Fuse and these existing methods did not use the same training dataset, we used the same independent dataset for a fair comparison of performances. We submitted the independent dataset directly to the web servers to obtain the prediction performances. The PUP-Fuse achieved the highest performance, as shown in Table 3, with a Sens of 0.59, a Spec of 0.91, an Acc of 0.82, and an MCC of 0.55. The PUP-Fuse provided 10–20% higher MCC than the other existing models, demonstrating the superiority of the PUP-Fuse over the existing predictors. The superiority of PUP-Fuse could result from a linear combination of the five ML probability scores evaluated by the five different encodings. Note that all the encodings contribute to prediction performance.

## 3. Materials and Methods

### 3.1. Data Collection and Processing

The datasets were retrieved and taken from the publication of the PupDB database [21]. The experimentally identified lysine pupylation sites were treated as positive samples, while all existing lysine residues that were not experimentally confirmed as the sites of pupylation in those proteins were treated as non-pupylation sites or negative samples. After deleting 80% similar sequences using CD-HIT [25], we preserved 233 pupylated proteins with 273 positive and 3280 negative sites. In the PupDB dataset, the ratio of the positive to negative samples (~1:12) is very unbalanced, which would obstruct the training model. Thus, a balanced dataset with a positive-to-negative ratio of 1:1 (186 of positive sites and negative 186 sites) was composed by randomly excluding the negative samples. The independent dataset consisting of 87 experimentally verified pupylation sites and 191 putative non-pupylation sites was randomly extracted from the dataset to test the various predictors. The curated datasets are summarized in Table 4.

### 3.2. Encoding Scheme

#### 3.2.1. pbCKSAAP

The pbCKSAAP method is widely investigated in previous studies [20,26,27,28]. The k-spaced residue pair could be defined as $p_{a}\;\left\{k\right\}\;p_{b}\left(a,\;b\;=\;1,2,\ldots ,20\right)$, where $p_{a}$ and $p_{b}$ show two residues of 20 types of amino acids. While k = 0, $p_{a}\;\left\{k\right\}\;p_{b}\;$represents a dipeptide and considers a number of 400 (=20×20) dipeptides. In this study k = 0,1,2,3,4 were considered ($i.e.,\;k_{max}\;=\;4$). Accordingly, the feature vector from each positive/negative sample has a dimension of 200 (=400×5 ). In this process, PSI-BLAST searched each protein sequence to produce a profile (i.e., PSSM matrix) with respect to the NCBI NR90 database (December 2010 version). For the inclusion of new sequences, the iteration time and e-value limit were set, respectively, to 3 and $1.0\times 10^{-4}$.

If residue pair $p_{a}\;\left\{k\right\}\;p_{b}$ performs between the positions t and t+k+1 in the PSSM matrix, the frequency scores could be generated as follows:

(1) $$S_{a,b}=\;\sum_{i=1}^{N}max\left\{min\left\{\mathrm{PSSM}\left(t,p_{a}\right),\;\mathrm{PSSM}\;\left(t+k+1,p_{b}\right)\right\},0\right\}$$

where $\mathrm{PSSM}\left(t,p_{a}\right)\;$denotes the amino acid score $p_{a}$ at the $t^{th}$ of PSSM in a row position, PSSM $\left(t+k+1,p_{b}\right)$ exists for an amino acid score of $p_{b}$ at ${\left(t+k+1\right)}^{th}\;$of PSSM in a row position. The pupylation/non-pupylation site appears N times. Moreover, we normalize $S_{a,b}$ using the formulation below:

(2) $${S^{\prime }}_{a,b}=\frac{S_{a,b}}{L\;-\;k-1}$$

where *L* stands for the total sequence fragment length, i.e., the size of a window is *L*. We have used the pbCKSAAP encoding scheme to create a 2000-dimensional feature vector for any positive/negative sample.

#### 3.2.2. CKSAAP Encoding

The CKSAAP encoding is widely used for representing sequence motifs [26,27,29]. If a sequence fragment is composed of 57 windows and 20 types of residues, it contains 400 (=20 × 20) types of residue pairs (i.e., AA, AC, AD, …) for every single *k*, where *k* signifies the space between two amino acids. In this work, the optimal *k_max_* was set to 4 to generate 2000 (=20 × (*k_max_* + 1) × 20)-dimensional feature vectors for a single sequence.

#### 3.2.3. Binary Encoding

Twenty types of amino acids can encode with the sequence window to generate the binary feature vectors [30,31]. By binary encoding, a 1140 (=20 × 57)-dimensional feature vector was calculated for a window sequence.

#### 3.2.4. TPC Encoding

The TPC encoding scheme implements a three-amino acid-fixed length of composition [29] to generate tri-amino acids composition with 8000 (=20 × 20 × 20)-dimensional feature vectors.

#### 3.2.5. AAI Encoding

The AAI encoding scheme uses the amino acid properties [32]. We selected the top 15 instructive amino acid indices after assessing different physicochemical and biological properties of amino acids (Table S2). The AAI encoding generates 855 (=57 × 15)-dimensional feature vectors.

#### 3.2.6. Feature Selection

Feature selection is a key step to eliminate unrelated features and to improve predictive performance. All the features are not equally important, or even some of them are noisy and have adverse effects on performance [15,20]. We used ChiSquaredAttributeEval and Ranker evaluation tools of WEKA [33] to select the features that are relevant to pupylation sites.

The chi-squared (χ^2^) test is a standard statistical test that analyzes the variance of the expected distribution, assuming that the presence of a given function is independent of the class value. Details in the χ^2^ feature selection process can be found elsewhere [20].

#### 3.2.7. Classification Method

Random forest algorithms are based on the classification and regression trees (CART) techniques [26,31,34]. It raises numerous trees of classification or regression that are called “forests”. Each tree is constructed using a deterministic algorithm, and due to two factors, the trees are different. The best separation is initially chosen from a random subset of the predictors at each node. In addition, a bootstrap observation sample is used to construct each tree. The overall prediction is then calculated as the average of all the trees.

We used KNN and SVM to compare with the RF classifier employed by PUP-Fuse. SVM is being widely used in protein bioinformatics [35,36,37]. For making a proper binary prediction, a kernel radial basis function (RBF) with the LIBSVM 2019 package (http://www.csie.ntu.edu.tw/~cjlin/libsvm/ (accessed date: 11 September 2019)) was applied to the training and independent datasets [38]. For tuning parameters, C and *γ* were maximized based on the training dataset by using the LIBSVM grid search strategy. The grid search strategy was carried based on 10-fold cross-validation tests to find the optimal C and *γ* ∈ [2^−7^, 2^−6^, …, 2^8^]. The KNN is a supervised ML algorithm that solves both classification and regression problems. We used the KNN algorithm of the R package to classify positive and negative samples at (https://cran.r-project.org (accessed date: 11 September 2019)).

#### 3.2.8. Feature Integration

Features were typically integrated to improve the prediction performance. We linearly combined the ML scores evaluated by the five encoding schemes: AAI, Binary, TPC, CKSAAP, and pbCKSAAP, with the formula as follows:

(3) $$\;Cl=\;\sum_{i=1}^{n}\left(w_{i\;}\;s_{i}\right)$$

where *Cl* is the linear combination of the ML scores, $w_{i\;}$ and $s_{i}$ are the weight coefficient and score for each encoding scheme *i*. The total of $w_{i\;}$ equals to 1. The linear combination model of the ML scores calculated by the five encoding schemes is named the PUP-Fuse. The above feature integration model is widely used in different bioinformatics tasks [27,39,40,41].

#### 3.2.9. Model Evaluation

In this analysis, 10-fold cross-validation was chosen to test the predictor proposed [31,42,43,44,45,46,47,48,49,50,51,52,53,54,55]. Seven measures were used to evaluate the proposed predictor: sensitivity (Sens), specificity (Spec), accuracy (Acc), Matthews coefficient of correlation (MCC) [30,46,56,57,58,59,60,61,62,63], precision and recall. The formulas are defined as follows:

(4) $$\mathrm{Sens}\;=\;\frac{\mathrm{TP}}{\mathrm{TP}\;+\;\mathrm{FN}}$$

(5) $$\mathrm{Spec}\;=\;\frac{\mathrm{TN}}{\mathrm{TN}\;+\;\mathrm{FP}}$$

(6) $$\mathrm{Acc}\;=\;\frac{\mathrm{TP}\;+\;\mathrm{TN}}{\mathrm{TP}\;+\;\mathrm{TN}\;+\;\mathrm{FP}\;+\;\mathrm{FN}}$$

(7) $$\mathrm{MCC}\;=\;\frac{\mathrm{TP}\;\times \;\mathrm{TN}\;-\;\mathrm{FP}\;\times \;\mathrm{FN}}{\sqrt{\left(\mathrm{TP}\;+\;\mathrm{FN}\right)\times \left(\mathrm{TN}\;+\;\mathrm{FP}\right)\times \left(\mathrm{TP}\;+\;\mathrm{FP}\right)\times \left(\mathrm{TN}\;+\;\mathrm{FN}\right)}}$$

(8) $$\mathrm{Precision}=\frac{\mathrm{TP}}{\mathrm{TP}+\mathrm{FP}}$$

(9) $$\mathrm{Recall}=\frac{\mathrm{TP}}{\mathrm{TP}+\mathrm{FN}}$$

where TP, FP, TN, and FN represent, respectively, the numbers of true positive, false-positive, true negative, and false-negative samples. Moreover, the area under the curve value (AUC) is evaluated from the receiver operating characteristic (ROC) curve by pROC package at https://cran.r-project.org/web/packages/pROC/ (accessed date: 11 September 2019), and the area under the precision curve (auPRC) is calculated to access the overall predictive performance.

## 4. Conclusions

The PUP-Fuse was developed for better prediction of pupylation sites. The PUP-Fuse was the RF model that integrated the five types of encoding schemes to consider various sequence patterns around protein pupylation sites. Then, the chi-squared test was used as the feature selection method. Performance evaluated by the training and independent tests clearly demonstrated the advantage of the PUP-Fuse over the existing models. The performances of PUP-Fuse are assessed based on the independent test dataset and compared with other existing methods, concluding that the predictive performance of PUP-Fuse is better than other existing methods. The comparison between different classifiers shows that the chi-squared feature selection algorithm optimized the curated feature vectors and RF-based model superior to other classifiers in predicting pupylation sites. Additionally, we found that the integration of successive ML scores by using a linear regression model advances the prediction performances. The web implementation of the PUP-Fuse with curated datasets are freely available for users at http://kurata14.bio.kyutech.ac.jp/PUP-Fuse/ (accessed date: 11 September 2019).

## Figures and Tables

**Table 1:** Prediction performance comparison among the single encoding-employing models and their combined models without any feature selection on the training dataset.

Encoding Method	Sens	Spec	Acc	MCC	AUC	*p*-Value
AAI	0.482	0.811	0.651	0.313	0.697	<0.01
Binary	0.510	0.810	0.661	0.331	0.703	<0.01
pbCKSAAP	0.782	0.800	0.800	0.590	0.908	0.034
TPC	0.770	0.801	0.791	0.574	0.877	0.021
CKSAAP	0.773	0.805	0.789	0.583	0.895	0.038
PUP-Fuse	0.802	0.820	0.811	0.623	0.912	

The PUP-Fuse is the linear combination of the RF score estimated by AAI, Binary, pbCKSAAP, CKSAAP, and TPC encodings and their weight coefficient are 0.1, 0.1, 0.3, 0.3, and 0.2, respectively.

**Table 2:** Performance comparison among the single encoding-employing models and their combined models with feature selection on the training dataset.

Encoding Method	Sens	Spec	Acc	MCC	AUC	*p*-Value
AAI	0.410	0.854	0.626	0.294	0.724	<0.01
Binary	0.417	0.855	0.629	0.305	0.731	<0.01
pbCKSAAP	0.831	0.827	0.829	0.658	0.929	0.031
TPC	0.754	0.827	0.789	0.582	0.878	<0.01
CKSAAP	0.822	0.825	0.824	0.646	0.911	<0.026
PUP-Fuse	0.886	0.881	0.884	0.768	0.956	

The PUP-Fuse is the linear combination of the RF score estimated by AAI, Binary, pbCKSAAP, CKSAAP, and TPC encodings and their weight coefficient are 0.1, 0.1, 0.3, 0.3, and 0.2, respectively.

**Table 3:** Performance comparison of the PUP-Fuse with the four existing methods on the independent dataset.

Methods	Sens	Spec	Acc	MCC
iPUP	0.40	0.88	0.73	0.32
GPS-PUP	0.21	0.89	0.68	0.13
PUPS	0.17	0.89	0.67	0.08
pbPUP	0.48	0.82	0.79	0.45
PUP-Fuse	0.59	0.91	0.82	0.55

The threshold values of iPUP, GPS-PUP, PUPS, and pbPUP were set to show high specificity (90%) in their corresponding webservers.

**Table 4:** The number of pupylated proteins and pupylation sites.

	Training	Independent
Pupylated protein	162	71
Pupylated lysine	186	87
Non-pupylated lysine	186	191

## Data Availability

All the data is available at http://kurata14.bio.kyutech.ac.jp/PUP-Fuse/ (accessed date: 11 September 2019).

## Supplementary Materials

Supplementary materials can be found at https://www.mdpi.com/1422-0067/22/4/2120/s1. Figure S1: Comparison of 1:1, 1:2, and 1:all ratios of positive-to-negative samples on training dataset; Figure S2: AUC values for different window sizes based on 10-fold cross-validation tests; Figure S3: AUC value with respect to selected features for the five encoding schemes; Table S1: Prediction performance after the removal of 30% sequence redundancy on the training dataset; Table S2: Fifteen types of AAI properties used in this study.

## References

[1] Li T., Chen Y., Li T., Jia C. (2018). Recognition of Protein Pupylation Sites by Adopting Resampling Approach. Molecules.

[2] Alhuwaider A.A.H., Truscott K.N., Dougan D.A. (2018). Pupylation of PafA or Pup inhibits components of the Pup-Proteasome System. FEBS Lett..

[3] Delley C.L., Striebel F., Heydenreich F.M., Ozcelik D., Weber-Ban E. (2012). Activity of the mycobacterial proteasomal ATPase Mpa is reversibly regulated by pupylation. J. Biol. Chem..

[4] Burns K.E., Darwin K.H. (2012). Pupylation: Proteasomal targeting by a protein modifier in bacteria. Methods Mol. Biol..

[5] Striebel F., Imkamp F., Ozcelik D., Weber-Ban E. (2014). Pupylation as a signal for proteasomal degradation in bacteria. Biochim. Biophys. Acta.

[6] Burns K.E., Darwin K.H. (2010). Pupylation versus ubiquitylation: Tagging for proteasome-dependent degradation. Cell Microbiol..

[7] Barandun J., Delley C.L., Weber-Ban E. (2012). The pupylation pathway and its role in mycobacteria. BMC Biol..

[8] Poulsen C., Akhter Y., Jeon A.H., Schmitt-Ulms G., Meyer H.E., Stefanski A., Stuhler K., Wilmanns M., Song Y.H. (2010). Proteome-wide identification of mycobacterial pupylation targets. Mol. Syst. Biol..

[9] Imkamp F., Rosenberger T., Striebel F., Keller P.M., Amstutz B., Sander P., Weber-Ban E. (2010). Deletion of dop in Mycobacterium smegmatis abolishes pupylation of protein substrates in vivo. Mol. Microbiol..

[10] Mukherjee S., Orth K. (2008). Microbiology. A protein pupylation paradigm. Science.

[11] Hecht N., Gur E. (2015). Development of a fluorescence anisotropy-based assay for Dop, the first enzyme in the pupylation pathway. Anal. Biochem..

[12] Xu X., Niu Y., Liang K., Shen G., Cao Q., Yang Y. (2016). Analysis of pupylation of Streptomyces hygroscopicus 5008 in vitro. Biochem. Biophys. Res. Commun..

[13] Fascellaro G., Petrera A., Lai Z.W., Nanni P., Grossmann J., Burger S., Biniossek M.L., Gomez-Auli A., Schilling O., Imkamp F. (2016). Comprehensive Proteomic Analysis of Nitrogen-Starved Mycobacterium smegmatis Deltapup Reveals the Impact of Pupylation on Nitrogen Stress Response. J. Proteome Res..

[14] Chen X., Li C., Wang L., Liu Y., Li C., Zhang J. (2016). The Mechanism of Mycobacterium smegmatis PafA Self-Pupylation. PLoS ONE.

[15] Nan X., Bao L., Zhao X., Zhao X., Sangaiah A.K., Wang G.G., Ma Z. (2017). EPuL: An Enhanced Positive-Unlabeled Learning Algorithm for the Prediction of Pupylation Sites. Molecules.

[16] Singh V., Sharma A., Dehzangi A., Tsunoda T. (2020). PupStruct: Prediction of Pupylated Lysine Residues Using Structural Properties of Amino Acids. Genes.

[17] Liu Z., Ma Q., Cao J., Gao X., Ren J., Xue Y. (2011). GPS-PUP: Computational prediction of pupylation sites in prokaryotic proteins. Mol. Biosyst..

[18] Tung C.W. (2013). Prediction of pupylation sites using the composition of k-spaced amino acid pairs. J. Theor. Biol..

[19] Chen X., Qiu J.D., Shi S.P., Suo S.B., Liang R.P. (2013). Systematic analysis and prediction of pupylation sites in prokaryotic proteins. PLoS ONE.

[20] Hasan M.M., Zhou Y., Lu X., Li J., Song J., Zhang Z. (2015). Computational Identification of Protein Pupylation Sites by Using Profile-Based Composition of k-Spaced Amino Acid Pairs. PLoS ONE.

[21] Tung C.W. (2012). PupDB: A database of pupylated proteins. BMC Bioinform..

[22] Vacic V., Iakoucheva L.M., Radivojac P. (2006). Two Sample Logo: A graphical representation of the differences between two sets of sequence alignments. Bioinformatics.

[23] Hasan M.M., Rashid M.M., Khatun M.S., Kurata H. (2019). Computational identification of microbial phosphorylation sites by the enhanced characteristics of sequence information. Sci. Rep..

[24] Hasan M.M., Yang S., Zhou Y., Mollah M.N. (2016). SuccinSite: A computational tool for the prediction of protein succinylation sites by exploiting the amino acid patterns and properties. Mol. Biosyst..

[25] Huang Y., Niu B., Gao Y., Fu L., Li W. (2010). CD-HIT Suite: A web server for clustering and comparing biological sequences. Bioinform..

[26] Hasan M.M., Khatun M.S., Kurata H. (2020). iLBE for Computational Identification of Linear B-cell Epitopes by Integrating Sequence and Evolutionary Features. Genom. Proteom. Bioinform..

[27] Khatun M.S., Hasan M.M., Kurata H. (2019). PreAIP: Computational Prediction of Anti-inflammatory Peptides by Integrating Multiple Complementary Features. Front. Genet..

[28] Hasan M.M., Khatun M.S., Mollah M.N.H., Yong C., Guo D. (2017). A systematic identification of species-specific protein succinylation sites using joint element features information. Int. J. Nanomed..

[29] Chen Y.Z., Tang Y.R., Sheng Z.Y., Zhang Z. (2008). Prediction of mucin-type O-glycosylation sites in mammalian proteins using the composition of k-spaced amino acid pairs. BMC Bioinform..

[30] Charoenkwan P., Yana J., Nantasenamat C., Hasan M.M., Shoombuatong W. (2020). iUmami-SCM: A Novel Sequence-Based Predictor for Prediction and Analysis of Umami Peptides Using a Scoring Card Method with Propensity Scores of Dipeptides. J. Chem. Inf. Model..

[31] Charoenkwan P., Nantasenamat C., Hasan M.M., Shoombuatong W. (2020). Meta-iPVP: A sequence-based meta-predictor for improving the prediction of phage virion proteins using effective feature representation. J. Comput. Aided Mol. Des..

[32] Kawashima S., Pokarowski P., Pokarowska M., Kolinski A., Katayama T., Kanehisa M. (2008). AAindex: Amino acid index database, progress report 2008. Nucleic Acids Res..

[33] Frank E., Hall M., Trigg L., Holmes G., Witten I.H. (2004). Data mining in bioinformatics using Weka. Bioinformatics.

[34] Khatun S., Hasan M., Kurata H. (2019). Efficient computational model for identification of antitubercular peptides by integrating amino acid patterns and properties. FEBS Lett..

[35] Khatun M.S., Hasan M.M., Shoombuatong W., Kurata H. (2020). ProIn-Fuse: Improved and robust prediction of proinflammatory peptides by fusing of multiple feature representations. J. Comput. Aided Mol. Des..

[36] Manavalan B., Basith S., Shin T.H., Wei L., Lee G. (2019). AtbPpred: A Robust Sequence-Based Prediction of Anti-Tubercular Peptides Using Extremely Randomized Trees. Comput. Struct. Biotechnol. J..

[37] Zhang D., Xu Z.C., Su W., Yang Y.H., Lv H., Yang H., Lin H. (2020). iCarPS: A computational tool for identifying protein carbonylation sites by novel encoded features. Bioinformatics.

[38] Chang C.C., Lin C.J. (2011). LIBSVM: A Library for Support Vector Machines. Acm. Trans. Intel. Syst. Tec..

[39] Hasan M.M., Alam M.A., Shoombuatong W., Kurata H. (2021). IRC-Fuse: Improved and robust prediction of redox-sensitive cysteine by fusing of multiple feature representations. J. Comput. Aided Mol. Des..

[40] Hasan M.M., Manavalan B., Khatun M.S., Kurata H. (2020). i4mC-ROSE, a bioinformatics tool for the identification of DNA N4-methylcytosine sites in the Rosaceae genome. Int. J. Biol. Macromol..

[41] Hasan M.M., Khatun M.S., Kurata H. (2019). Large-Scale Assessment of Bioinformatics Tools for Lysine Succinylation Sites. Cells.

[42] Ho Thanh Lam L., Le N.H., Van Tuan L., Tran Ban H., Nguyen Khanh Hung T., Nguyen N.T.K., Huu Dang L., Le N.Q.K. (2020). Machine Learning Model for Identifying Antioxidant Proteins Using Features Calculated from Primary Sequences. Biology.

[43] Hasan M.M., Kurata H. (2018). GPSuc: Global Prediction of Generic and Species-specific Succinylation Sites by aggregating multiple sequence features. PLoS ONE.

[44] Khatun M.S., Shoombuatong W., Hasan M.M., Kurata H. (2020). Evolution of Sequence-based Bioinformatics Tools for Protein-protein Interaction Prediction. Curr. Genom..

[45] Le N.Q.K., Do D.T., Hung T.N.K., Lam L.H.T., Huynh T.T., Nguyen N.T.K. (2020). A Computational Framework Based on Ensemble Deep Neural Networks for Essential Genes Identification. Int. J. Mol. Sci..

[46] Manavalan B., Hasan M.M., Basith S., Gosu V., Shin T.H., Lee G. (2020). Empirical Comparison and Analysis of Web-Based DNA N (4)-Methylcytosine Site Prediction Tools. Mol. Ther. Nucleic Acids.

[47] Charoenkwan P., Yana J., Schaduangrat N., Nantasenamat C., Hasan M.M., Shoombuatong W. (2020). iBitter-SCM: Identification and characterization of bitter peptides using a scoring card method with propensity scores of dipeptides. Genomics.

[48] Charoenkwan P., Nantasenamat C., Hasan M.M., Shoombuatong W. (2020). iTTCA-Hybrid: Improved and robust identification of tumor T cell antigens by utilizing hybrid feature representation. Anal. Biochem..

[49] Charoenkwan P., Kanthawong S., Nantasenamat C., Hasan M.M., Shoombuatong W. (2020). iDPPIV-SCM: A Sequence-Based Predictor for Identifying and Analyzing Dipeptidyl Peptidase IV (DPP-IV) Inhibitory Peptides Using a Scoring Card Method. J. Proteome. Res..

[50] Charoenkwan P., Kanthawong S., Nantasenamat C., Hasan M.M., Shoombuatong W. (2020). iAMY-SCM: Improved prediction and analysis of amyloid proteins using a scoring card method with propensity scores of dipeptides. Genomics.

[51] Charoenkwan P., Anuwongcharoen N., Nantasenamat C., Hasan M.M., Shoombuatong W. (2020). In silico approaches for the prediction and analysis of antiviral peptides: A review. Curr. Pharm. Des..

[52] Manavalan B., Basith S., Shin T.H., Lee G. (2020). Computational prediction of species-specific yeast DNA replication origin via iterative feature representation. Brief. Bioinform..

[53] Basith S., Manavalan B., Shin T.H., Lee G. (2019). SDM6A: A Web-Based Integrative Machine-Learning Framework for Predicting 6mA Sites in the Rice Genome. Mol. Ther. Nucleic Acids.

[54] Basith S., Manavalan B., Shin T.H., Lee G. (2018). iGHBP: Computational identification of growth hormone binding proteins from sequences using extremely randomised tree. Comput. Struct. Biotechnol. J..

[55] Hasan M.M., Shoombuatong W., Kurata H., Manavalan B. (2021). Critical evaluation of web-based DNA N6-methyladenine site prediction tools. Brief. Funct. Genom..

[56] Basith S., Manavalan B., Hwan Shin T., Lee G. (2020). Machine intelligence in peptide therapeutics: A next-generation tool for rapid disease screening. Med. Res. Rev..

[57] Manavalan B., Basith S., Shin T.H., Wei L., Lee G. (2019). mAHTPred: A sequence-based meta-predictor for improving the prediction of anti-hypertensive peptides using effective feature representation. Bioinformatics.

[58] Wei L., He W., Malik A., Su R., Cui L., Manavalan B. (2020). Computational prediction and interpretation of cell-specific replication origin sites from multiple eukaryotes by exploiting stacking framework. Brief. Bioinform..

[59] Su R., He L., Liu T., Liu X., Wei L. (2020). Protein subcellular localization based on deep image features and criterion learning strategy. Brief. Bioinform..

[60] Ning Q., Ma Z., Zhao X., Yin M. (2020). SSKM_Succ: A novel succinylation sites prediction method incorprating K-means clustering with a new semi-supervised learning algorithm. IEEE/ACM Trans. Comput. Biol. Bioinform..

[61] Ning Q., Yu M., Ji J., Ma Z., Zhao X. (2019). Analysis and prediction of human acetylation using a cascade classifier based on support vector machine. BMC Bioinform..

[62] Hasan M.M., Basith S., Khatun M.S., Lee G., Manavalan B., Kurata H. (2020). Meta-i6mA: An interspecies predictor for identifying DNA N6-methyladenine sites of plant genomes by exploiting informative features in an integrative machine-learning framework. Brief. Bioinform..

[63] Hasan M.M., Schaduangrat N., Basith S., Lee G., Shoombuatong W., Manavalan B. (2020). HLPpred-Fuse: Improved and robust prediction of hemolytic peptide and its activity by fusing multiple feature representation. Bioinformatics.

