# Supplementary material for: PUP-Fuse: Prediction of Protein Pupylation Sites by Integrating Multiple Sequence Representations
Source: Int J Mol Sci. 2021 Feb 20;22(4):2120. doi: 10.3390/ijms22042120 (PMC7924619; doi:10.3390/ijms22042120)
Supplement: Supplementary file 1 [file ijms-22-02120-s001.pdf]

Supplementary information

PUP-Fuse: Prediction of Protein Pupylation Sites by Integrating Multiple Sequence Representations

Firda Nurul Auliah[1], Andi Nur Nilamyani[1], Watshara Shoombuatong[2], Md Ashad Alam[3], Md Mehedi Hasan[1,4], and Hiroyuki Kurata[1*]

Table S1. Prediction performance after the removal of 30% sequence redundancy on the training dataset.

Encoding method	Sens	Spec	Acc	MCC	AUC
AAI	0.475	0.810	0.643	0.310	0.659
Binary	0.502	0.805	0.654	0.329	0.691
pbCKSAAP	0.769	0.815	0.792	0.585	0.880
TPC	0.761	0.811	0.786	0.580	0.866
CKSAAP	0.764	0.810	0.787	0.577	0.872
Pup-Fuse	0.781	0.824	0.803	0.615	0.903

The PUP-Fuse is the linear combination of the RF scores estimated by AAI, Binary, pbCKSAAP, CKSAAP, and TPC encodings and their weight coefficient are 0.1, 0.1, 0.4, 0.25, and 0.15 respectively.

Table S2. Fifteen types of AAI properties used in this study

AAI_ID	A	R	N	D	C	Q	E	G	H	I	L	K	M	F	P	S	T	W	Y
JANJ780103	15.	67.	49.	50.	5.	56.	55.	10.	34.	13.	16.	85.	20.	10.	45.	32.	32.	17.	41.
KANM800102	0.81	0.85	0.62	0.71	1.17	0.98	0.53	0.88	0.92	1.48	1.24	0.77	1.05	1.20	0.61	0.92	1.18	1.18	1.23
GEIM800107	0.91	0.99	0.72	0.74	1.12	0.90	0.41	0.91	1.01	1.29	1.23	0.86	0.96	1.26	0.65	0.93	1.05	1.15	1.21
GOLD730102	88.3	181.2	125.1	110.8	112.4	148.7	140.5	60.0	152.6	168.5	168.5	175.6	162.2	189.0	122.2	88.7	118.2	227.0	193.0
JOND920102	100.	83.	104.	86.	44.	84.	77.	50.	91.	103.	54.	72.	93.	51.	58.	117.	107.	25.	50.
FINA910102	1.	0.70	1.	1.70	1.	1.	1.70	1.30	1.	1.	1.	0.70	1.	1.	13.	1.	1.	1.	1.
GEIM800108	0.91	1.	1.64	1.40	0.93	0.94	0.97	1.51	0.90	0.65	0.59	0.82	0.58	0.72	1.66	1.23	1.04	0.67	0.92
FAUJ880104	2.87	7.82	4.58	4.74	4.47	6.11	5.97	2.06	5.23	4.92	4.92	6.89	6.36	4.62	4.11	3.97	4.11	7.68	4.73
GEIM800105	0.84	1.04	0.66	0.59	1.27	1.02	0.57	0.94	0.81	1.29	1.10	0.86	0.88	1.15	0.80	1.05	1.20	1.15	1.39
FASG760101	89.09	174.20	132.12	133.10	121.15	146.15	147.13	75.07	155.16	131.17	131.17	146.19	149.21	165.19	115.13	105.09	119.12	204.24	181.19
FASG760102	297.	238.	236.	270.	178.	185.	249.	290.	277.	284.	337.	224.	283.	284.	222.	228.	253.	282.	344.
GEIM800102	1.13	1.09	1.06	0.94	1.32	0.93	1.20	0.83	1.09	1.05	1.13	1.08	1.23	1.01	0.82	1.01	1.17	1.32	0.88
JANJ780102	51.	5.	22.	19.	74.	16.	16.	52.	34.	66.	60.	3.	52.	58.	25.	35.	30.	49.	24.
MIYS990104	-0.04	0.07	0.13	0.19	-0.38	0.14	0.23	0.09	-0.04	-0.34	-0.37	0.33	-0.30	-0.38	0.19	0.12	0.03	-0.33	-0.29
PUNT030101	-0.17	0.37	0.18	0.37	-0.06	0.26	0.15	0.01	-0.02	-0.28	-0.28	0.32	-0.26	-0.41	0.13	0.05	0.02	-0.15	-0.09

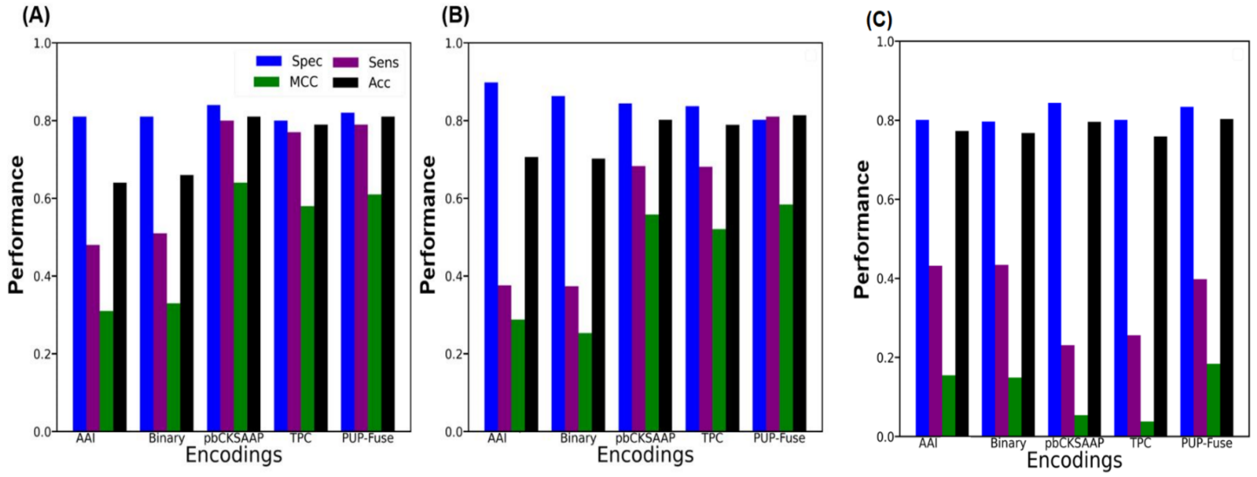

Figure S1. Comparison of 1:1, 1:2, and 1:all ratios of positive-to-negative samples on training dataset.

(A) 1:1 ratio. (B)1:2 ratio. (C) 1:all ratio.

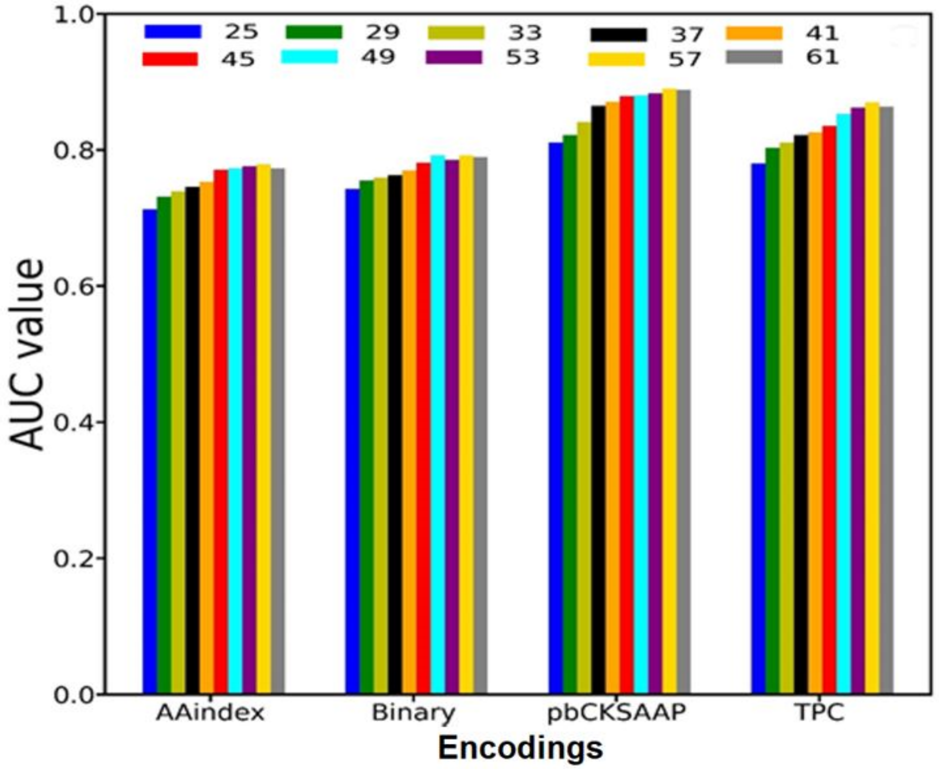

Figure S2. AUC values for different window sizes based on 10-fold cross-validation tests.

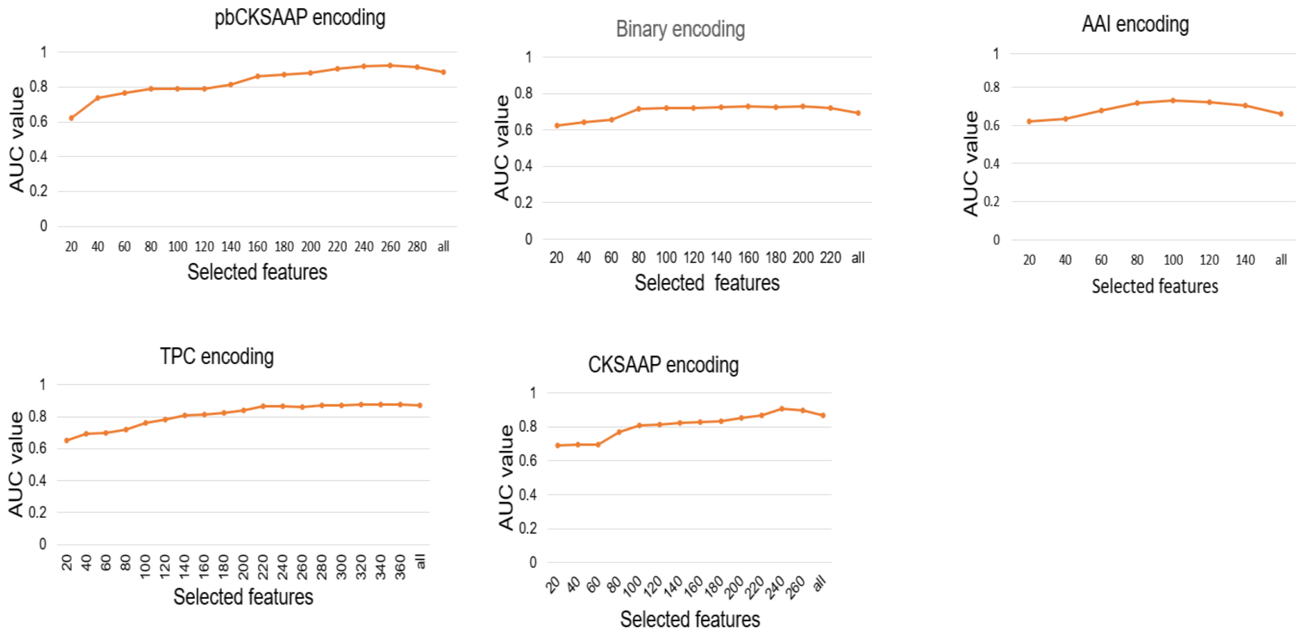

Figure S3. AUC value with respect to selected features for the five encoding schemes.
